# Interprofessional collaboration in the home care setting: perspectives of people receiving home care, relatives, nurses, general practitioners, and therapists—results of a qualitative analysis

**DOI:** 10.1186/s12875-024-02313-8

**Published:** 2024-03-04

**Authors:** Uta Sekanina, Britta Tetzlaff, Ana Mazur, Tilman Huckle, Anja Kühn, Richard Dano, Carolin Höckelmann, Martin Scherer, Katrin Balzer, Sascha Köpke, Eva Hummers, Christiane Müller

**Affiliations:** 1 Department of General Practice, University Medical Center Goettingen, Humboldtallee 38, 37073 Göttingen, Germany; 2 Department of General Practice and Primary Care, University Medical Center Hamburg- Eppendorf, Martinistr. 52, 20246 Hamburg, Germany; 3 Nursing Research Unit, Institute for Social Medicine and Epidemiology, University of Lübeck, Ratzeburger Allee 160, 23562 Lübeck, Germany; 4 Institute of Nursing Science, Medical Faculty, University of Cologne, University Hospital Cologne, Gleueler Str. 176-178, 50935 Köln, Germany

**Keywords:** Interprofessional collaboration, Home healthcare, Qualitative research, Health professions, People receiving home care, Relatives

## Abstract

**Background:**

About one million people in need of home care in Germany are assisted by 15,400 home care services. Home healthcare is mostly a complex endeavour because interprofessional collaboration is often challenging. This might negatively impact patient safety. The project *interprof* HOME aims to develop an interprofessional person-centred care concept for people receiving home care in a multistep approach. In one of the work packages we explored how people receiving home care, relatives, nurses, general practitioners, and therapists (physiotherapists, occupational therapists, and speech therapists) perceive collaboration in this setting.

**Methods:**

Semi-structured interviews were conducted with 20 people receiving home care and with 21 relatives. Additionally, we worked with nine monoprofessional focus groups involving nurses of home care services (*n* = 17), general practitioners (*n* = 14), and therapists (*n* = 21). The data were analysed by content analysis.

**Results:**

Three main categories evolved: “perception of interprofessional collaboration”, “means of communication”, and “barriers and facilitators”. People receiving home care and relatives often perceive little to no interprofessional collaboration and take over a significant part of the organisational coordination and information exchange. Interprofessional collaboration in steady care situations does exist at times and mostly occurs in coordination tasks. Contact and information exchange are rare, however, interprofessional personal encounters are sporadic, and fixed agreements and permanent contact persons are not standard. These trends increase with the complexity of the healthcare situation. Joint collaborations are often perceived as highly beneficial. Means of communications such as telephone, fax, or e-mail are used differently and are often considered tedious and time-consuming. No interprofessional formal written or electronic documentation system exists. Personal acquaintance and mutual trust are perceived as being beneficial, while a lack of mutual availability, limited time, and inadequate compensation hinder interprofessional collaboration.

**Conclusions:**

Interprofessional collaboration in home care occurs irregularly, and coordination often remains with people receiving home care or relatives. While this individual care set-up may work sufficiently well in low complex care situations, it becomes vulnerable to disruptions with increasing complexity. Close interactions, joint collaboration, and fixed means of communication might improve healthcare at home. The findings were integrated into the development of the person-centred interprofessional care concept *interprof* HOME.

**Trial registration:**

This study is registered on the International Clinical Trails registry platform ClinicalTrials.gov as NCT05149937 on 03/11/2021.

**Supplementary Information:**

The online version contains supplementary material available at 10.1186/s12875-024-02313-8.

## Background

Life expectancy in Germany increases continuously. The number of people in need of care steadily grows as well [1]. The majority (4.1 million) of the almost 5 million persons in need of care in 2021 received care in their own homes most often supported by their relatives [1]. The older the persons in need of care were, the more frequently a home care service was involved. At the end of 2021, about 15,400 home care services provided care to over one million people receiving home care [1].

In addition, relatives, general practitioners (GPs), physiotherapists, occupational therapists, and speech therapists are often involved in the care of people receiving home care (PRHC). Not much is known in Germany about the perspectives on interprofessional collaboration of all person groups that are mainly involved. A profound insight into their perspectives is essential to understand the current forms of collaboration in the home care setting.

According to the World Health Organization (WHO; 2010), “collaborative practice in healthcare occurs when multiple health workers from different professional backgrounds provide comprehensive services by working with patients, their families, carers and communities to deliver the highest quality of care across settings” [2]. Interprofessional collaboration enhances care quality by fostering a comprehensive approach considering diverse patient needs through the involvement of professionals from various disciplines [3, 4]. The international scientific literature has reported that interprofessional collaboration in home care is in need of improvement: The unclear definition of roles and responsibilities, accompanied by a lack of information sharing, reveals the problematic nature of working relationships between professional groups [5–7]. Furthermore, professionals often lack insight into the typical working processes of the other professionals in home care [8] and are not sufficiently aware of information needed to deliver good quality of care [9]. In addition, structural barriers regarding availability, documentation systems, and fragmentation of care due to geographical conditions are evident [8]. Moreover, international studies show an inhomogeneous picture of the perspectives of the involved groups: GPs and home care services complain about awkward, irregular, and unsatisfactory communication and documentation in the cooperation [10], while in another study, GPs rate the collaboration with physiotherapists and home services as very important and satisfactory [11]. GPs perceive themselves as the coordinators of care [7, 11, 12] because they often take responsibility for the medication and discuss diagnoses and test results [11]. Nurses rarely meet GPs or other professionals and are not always involved in medical processes unless complications arise [7]. Therapists performing home visits criticise the low level of exchange with GPs and home care services [13]. For the perspective of PRHC from an interprofessional collaboration, Careau et al. showed that to meet the needs of PRHC, PRHC and relatives should be considered as team members in a collaborative practice [14]. Although PRHC are generally satisfied with their care [7, 15], they only sometimes perceive common agreements between GPs and their own relatives. In particular, older people do not experience interprofessional collaboration in home care as such [16]. In Germany, there is currently a lack of comprehensive data regarding the perspective of interprofessional collaboration in the context of home care. These insights are essential for developing a sustainable and effective healthcare model for the future.

### Research question

How do PRHC, relatives, nurses from home care services, GPs, and members of therapy professions perceive the interprofessional collaboration in the home care setting?

## Methods

### Research design

This work is part of the exploratory mixed-methods study *interprof* HOME, which aims to develop an interprofessional person-centred care concept for the healthcare of PRHC in a stepwise process. The qualitative approach of this sub-study examined the subject-related perspective of PRHC, relatives, and professionals in home healthcare through semi-structured interviews with PRHC, relatives, and through focus groups with professionals. This reflects real-life situations and is close to natural surroundings [17, 18]. The data collection was conducted between January 2022 and July 2022. The eligibility criteria were defined in our study protocol [16].

### Sampling

Participants were recruited purposefully with regard to heterogeneous characteristics such as gender, age, residence, care provision, and relation to relatives. The participants of the focus groups were selected to vary in gender, residence, and work experience (see Table 1).

**Table 1:** Participants‘ demographics

Variables	PRHC* (*n* = 20)	Relatives (*n* = 21)	Nurses (*n* = 17)	GPs (*n* = 14)	Therapists** (*n* = 21)
Gender	Female (*n* = 14) Male (*n* = 6)	Female (*n* = 16) Male (*n* = 5)	Female (*n* = 13) Male (*n* = 4)	Female (*n* = 9) Male (*n* = 5)	Female (*n* = 15) Male (*n* = 6)
Age (years)	31–99	36–87	26–61	36–69	25–60
Residence	Rural (*n* = 13) Urban (*n* = 7)	Rural (*n* = 7) Urban (*n* = 14)	Rural (*n* = 4) Urban (*n* = 13)	Rural (*n* = 8) Urban (*n* = 6)	Rural (*n* = 4)** Urban (*n* = 16)****
Relation to PRHC		Partner (*n* = 10) Mother (*n* = 8) Father (*n* = 1) Son (*n* = 1) Others (*n* = 1)			
Care*** provision by	Relatives (*n* = 11) Nurses (*n* = 19) GP (*n* = 6)	Nurses (*n* = 20) GP (*n* = 6) Therapists**(*n* = 22)			
Care level*	2 (*n* = 11) 3 (*n* = 5) 4 (*n* = 2) 5 (*n* = 2)	2 (*n* = 2) 3 (*n* = 3) 4 (*n* = 10) 5 (*n* = 6)			
Work experience (years) Mean (years)			2–30 7.75	7–42 19.75	2–29 12.86
Home visits per week (number)			1-100	0–10	1–50

*classification 2 to 5, minimal to the most serious impairment of independence or ability, ** physio/occupational/ speech therapists; ***multiple answers possible; ****missing answer

### Methods of approach

The home care services and GPs were the initial channel through which we reached PRHC and relatives. The home care services and GPs were identified via local registers and invited by letter. We later contacted them again by phone. They were asked to inform their PRHC or relatives of their PRHC about the study, and if PRHC or relatives were interested, ask for written permission to forward the contact information to the researchers. In another pass, the consenting PRHC or relatives were contacted and again asked for participation in the interviews by the researchers. Furthermore, PRHC and relatives were approached by phone on the basis of internet-listed self-help groups. For the focus groups, local registered home care services, GPs, physiotherapists, occupational therapists, and speech therapists were invited by letter and phone. All participants received detailed written and oral information from a researcher before they signed the informed consent.

### Sample size

A sample size of 20 interviews with PRHC and 21 interviews with relatives of PRHC was sought across the four research centres considering different geographical aspects. All centres were located in cities: Hamburg and Cologne with over 1 million residents, and Göttingen and Lübeck with less than 250,000 residents. In all centres participants from both urban and rural environments were included. Three monoprofessional focus groups with about eight participants from each professional group (nurses, GPs, therapists) were planned to be conducted (nine focus groups altogether) in the regions of the four research centres. In the end, we interviewed 20 PRHC and 21 relatives of PRHC, and we worked with nine monoprofessional focus groups, three for each profession. Professionals had no connection with the interviewed PRHC or relatives and were not working together. Also, PRHC and relatives were not related to each other. A total of 17 nurses (two groups with five persons each, one group with seven persons), 14 GPs (two groups with four persons each, one group with six persons), and 21 therapists (three groups with seven persons each) participated in the focus groups (see Table 2).

**Table 2:** Number of participants in all research centres

	PRHC	relatives	nurses	GPs	therapists
Semi-structured interviews	20	21			
Focus group 1			5	4	7
Focus group 2			5	4	7
Focus group 3			7	6	4
Total	20	21	17	14	21

### Data collection

The guidelines for the semi-structured interviews and focus groups was based on the literature according to Helfferich [19]. A pilot interview led to adaptations. After an introductory question about typical daily healthcare, additional topics were addressed in the interviews, including experiences of the interviewees with interprofessional cooperation, person-centred care, changes due to the COVID-19 pandemic, and ideas and requests with regard to the ideal healthcare at home (see Supplementary Material 1). In the focus groups, the discussion targeted the personal experience with interprofessional care, collaboration in general, and communication with PRHC, relatives, and other professionals, and finally, ideas for optimising collaboration in home care (see Supplementary Material 2). We choose to work with focus groups to benefit from interactions within the group in addition to the individual perspectives of participants [20].

Due to the COVID-19 pandemic, the interviews were conducted by phone. The focus groups took place via video conferences. The mean length of interviews was 43 min, focus groups took on average 105 min.

Eight researchers (AM, US, BT, AMR, TH, AK, RD, and CH) conducted interviews and led focus groups. The researchers have various professional backgrounds: a physiotherapist with an academic degree in Public Health (AM), a paediatric registered nurse with an academic degree in Education for Health Professions (US), a registered occupational therapist with an academic degree in Occupational Therapy (BT), a registered nurse with an academic degree in Nursing Sciences (TH), a registered nurse with an academic degree in Nursing (AMR), a registered nurse with an academic degree in Health and Nursing Sciences (AK), a paediatric registered nurse with an academic degree in Health Service Research (RD), and a registered nurse with an academic degree in Nursing Science (CH). Researchers and study participants did not have prior contact.

Some researchers had already collaborated in the research projects *interprof* [21] and *interprof* ACT [22]. All researchers and instructing professors have several years of experience in qualitative research.

Interviews and focus groups were audio-taped and transcribed verbatim in German. Focus groups were recorded in writing. The quotes of the interviews were translated for this paper. Postscripts were written after the interviews to record location and time, as well as the mood of the PRHC or relatives, the atmosphere and significant aspects during the interview as additional information. Postscripts were used to verify congruence with the content of the interviews.

### Data analysis

The data analysis of the interviews (13 to 89 min) and focus groups (71 to 130 min) was conducted in compliance with the principles of qualitative content analysis [23] using MAXQDA2022 as a software tool.

The basis of the analysis was an a priori coding frame defined by the interview guideline. The coding frame was enhanced by codes that emerged inductively from the first interview with a relative. The codes were discussed by the evaluation group (AM, US, BT, TH, RD, and CH) until a common coding basis for the interviews of the PRHC and relatives was agreed. Interviews with the relatives were analysed by US, BT, RD, and CH, and interviews with the PRHC were analysed by AM and TH. After the joint analysis of six interviews, the original coding frame was refined to cover all topics or specific aspects that had not been detected before. The remaining 35 interviews were peer-reviewed, discrepancies were discussed, and modifications were agreed on in researchers’ dyads. For the analysis of the focus groups (BT, US), a coding frame was generated using the existing coding frame of relatives and PRHC as the initial basis. This existing coding frame was inductively refined. A matrix was used to elaborate differences and commonalities of the perspectives of all person groups (US), which was repeatedly discussed with CM and EH.

## Results

The analyses have resulted in the following coding frame considering the perspectives of all involved person groups (see Table 3):

**Table 3:** Coding frame

Main category	Subcategory
Perception of interprofessional collaboration	• No or rare interprofessional collaboration • Organisational coordination • Exchange of information o PRHC and relatives as mediators o Nurses as mediators o Direct exchange between professionals • Joint collaboration o Complementary work o Meetings o Handing-over of tasks
Means of communication	• Fax • Phone • Messenger service • E-mail • Written documentation
Barriers and facilitators of interprofessional collaboration	• Person-related factors o Being known o Recognition of expertise • Structural conditions o Availability o Financial and time constraints o Fragmentation of care

### Perception of interprofessional collaboration

The main category “perception of collaboration” contains subcategories uncovering a great diversity of perspectives between the groups and sometimes also within a group. While most PRHC and relatives perceive no interprofessional collaboration or cannot elaborate on it, many professionals perceive collaboration in very differentiated ways.

### No or rare interprofessional collaboration

The subcategory “No or rare collaboration” comprises aspects with regard to infrequent contact and information exchange between professionals. It was mentioned by some GPs and most PRHC and relatives:

*“The occupational therapist never said “I’m going to talk with the physiotherapist”. And the physiotherapist didn’t say either that he was going to talk with them. So, everyone just did their thing. And the general practitioner talked neither with her nor with him. The nursing service spoke neither with the therapists nor with the general practitioner.”* (relative: C1/44)^1^

Participants stated that, when everything is going well, no or infrequent interaction is often perceived as sufficient, and they do not require contact or exchange of information:

*“We don’t meet frequently or hear what the other is doing when all is going according to plan.”* (GP: C/18).

However, no or rare collaboration is often seen as insufficient and is critical for optimal care, as PRHC, relatives, and professionals described:

*“[…] Our experience every day is that because there are no consultations or contact, some areas in the end don’t receive the best possible treatment.”* (relative: D1/66).

### Organisational coordination

The PRHC, relatives, nurses, GPs, and therapists perceive organisational coordination, that is, the scheduling of appointments and the handling of prescriptions, as challenging but necessary. Appointments and prescriptions are mostly scheduled and handled by PRHC and relatives:

*“[…], whether I need to check the prescription for physiotherapy, but that’s not hard for me, and everyone can do something on their own.”* (PRHC: A2/194).

Some PRHC and relatives reported that it is important to actively coordinate appointments and prescriptions. Others feel a high organisational burden, especially when they have to handle incomplete or incorrectly filled forms. In general, participants of all groups perceive the coordination by PRHC and relatives as mostly effective:

*Well, […] in my experience, things [managing the appointments] work quite well when some relatives still live at home.”* (therapist: A/24).

Professionals stated that they also occasionally take over the coordination. In some cases, this contact is the only contact between the professionals, but it is a regular contact. While some PRHC are unaware of these interprofessional processes, relatives often perceive these proceedings as sufficient:

*“They also collaborate in a way that avoids having to reschedule appointments or that avoids overlapping visits (smiles slightly)”.* (relative: D4/32)

Challenges with regard to organisational coordination mainly arise between nurses and therapists because their organisational structures are different and coordination tools are lacking. While a few nurses complained that therapists receive priority in the scheduling of home visits, therapists mentioned difficulties that arise because the care schedules of the nurses are too fixed. The consequences of these challenges are overlapping appointments, loss of therapy time, or even cancelled therapy sessions. Some therapists reported that they directly coordinate appointments with nurses to prevent scheduling conflicts.

### Exchange of information

This subcategory contains information on current practices of information exchange. The participants of all professional groups and some of the relatives perceive the exchange of information as crucial for the interprofessional collaboration in general. They stated that they see a need for discussing therapy or further diagnostics. Often, little is known about the expertise of the other professions. A holistic view is desired, which needs exchange of information, and therapeutic treatment in the care-planning process of nurses is required as well. Some professionals perceive the exchange of information as enriching because it results in improved care. The actual task of information exchange is frequently viewed as depending on the persons involved:

*“Indeed, the consultations are crucial for us not to work against each other and also to gain some understanding for the respective other areas, in the end, and that strongly depends on the person involved, in my opinion.”* (therapist: D/42).

#### PRHC and relatives as mediators

Many professionals reported that most of the information concerning medical diagnostics or initiation of therapies is forwarded via PRHC or relatives. Similarly, many PRHC and relatives see themselves as information exchangers between professionals:

*“[…] I just managed the whole thing as a manager, as it were.”* (relative: C1/22).

In this context, some PRHC and relatives stated that they decide by themselves which information to provide:

*“Yes. What I don’t tell others remains a secret.”* (relative: D5/41).

Some professionals criticised that as a consequence, no trustworthy information is passed on. Because some therapists receive oral messages that are distorted or forgotten, they favour passing on information in writing.

#### Nurses as mediators

Nurses made clear that they often hold the role of information exchangers when PRHC or relatives are overburdened or no relatives are available:

*“Well, with some relatives, they are entirely in over their heads and the nursing service is just helpful for them. Much is organised by the nursing service.”* (GP: A/31).

In these cases, the provision of information via nurses is regarded as mandatory by GPs. This is also perceived to be the case by some nurses themselves. Similarly, some therapists address their concerns via nurses.

Nurses confirmed, although they are aware of this attributed role, they feel burdened when their concerns are not dealt with promptly and they repeatedly have to remind the GP or the office staff. However, some GPs criticised nurses because agreements are sometimes not kept or information is not passed on to the nurse who is in charge.

#### Direct exchange between professionals

Direct communication through personal interaction between professionals rarely takes place in usual healthcare situations in general. However, some nurses reported that GPs regularly contact them to exchange information. Moreover, some GPs pointed out that they have no problem to contact the nurse who is in charge. Therapists reported a mixed picture: Some enjoy good direct contact with nurses and GPs for many years, others observe that they are not being contacted by them directly. Especially when the care demand increases or becomes urgent, such as changes in wound conditions or medication prescriptions, professionals intensify their direct contacts and exchange or share more information:

*“If it’s something urgent, one of the therapists may call and draw attention to something. “Yes, I am concerned. This is quite serious after all, somehow”.* (GP: A/70)

Most professionals claim to be the ones initiating the contact:

*“[…], well, you need to initiate communication with the physiotherapist yourself, don’t you? I’ve never yet had the experience that this would work in the other direction. That they would initiate communication with you.”* (GP: C/20).

The PRHC and relatives affirmed that they often know when information is exchanged between professionals, or they are asked before the exchange and are satisfied with these arrangements. On the other hand, some PRHC only assume that GPs and nurses will share information. Some PRHC expressed that they do not wish professionals to discuss anything without their knowledge:

*“I don’t think nursing service 1 would consult the doctor behind my back. That would be outrageous.”* (PRHC: C4/57).

### Joint collaboration

The subcategory “Joint collaboration” contains data on collaboration in more or less complex care situations. Complex care situations are situations in which the medical, nursing, and therapeutic needs are high. This may be a chronic or long-term healthcare situation, situations in which the PRHC and relatives are overwhelmed, or situations in which the PRHC has no relatives. Less complex care situations are characterized by mainly coordinating and organisational tasks and/or simple medical, nursing and therapeutic tasks. In complex care situations, the professionals reported that they work more closely together. Professionals carry out bedside-teaching, joint meetings, or home visits to cope with challenges in the healthcare of the PRHC.

#### Complementary work

Hands-on support by therapists is reported especially in 24-hour nursing care. Nurses consider the collaboration with therapists as enriching for their nursing care and as improving the quality of care in general:

*“A physiotherapist can teach me how they go about helping the patient with some movements so that I can do this in the morning, at lunchtime, and in the evening, three times a day, and then progress is considerably sped up.”* (nurse: D/71).

Vice versa, nurses specified that they value it, when the therapists combine a swallowing therapy with assistance in meal ingestion:

*„Certainly, we can work even better if there actually is some food, we can then practice swallowing with the food.“* (therapist: A/64).

#### Meetings

Interprofessional meetings in person are a rarity and difficult to organise, but can facilitate and improve information exchange on care issues. Some nurses and therapists mentioned that they prefer to drop by at the GP practice for personal on-site talks and for immediate answers to health-related and organisational requests. These visits create an increased workload, and some home-service providers reported that they therefore charge the PRHC for this additional service:

*“Well, reordering the medication, fetching the prescription and getting the prescription filled at the drugstore. That would […] mean an additional flatrate of 50 Euro each time.”* (relative: A01/56).

Some GPs acknowledged that regular meetings with a nurse support the care. Out of personal commitment, one GP stated that he/she stops by in the office of the home-service provider, and another GP informed that he/she visits the home of the PRHC before office hours to meet the nurses during their morning routine.

Moreover, GPs reported that meetings with therapists range from marginal in-person contact to on-the-wing conversations with substantial exchanges and even regular digital case-conferences to discuss the progress of the PRHC and further joint action, such as changing bandages or wound management.

Interprofessional home visits are sometimes organised by the relatives, the PRHC themselves, or professionals. However, one GP suspected that the nurses attend GP-initiated interprofessional home visits only because the GP is a person of authority.

#### Handing-over of tasks

Some GPs leave information about the available support options for the nurses, such as relief and assistance services, and also care services. They commented that they value the nurses as an “*extended medical eye*” (GP: A/41) during their daily visits and often adopt their healthcare recommendations:

*“[…] Older practitioners who are still prepared to do home calls, who have known the patient for a long time and the […] have no clue, or only little knowledge of what is required. Some then say, “Okay, we’re going to proceed with this as you as the nurse recommend.”* (nurse: D/53).

*“It relieves me because the nursing service is like an extended medical eye. I cannot be there all the time. And the nurse sees the patient every day. […] And by and by, you get to know the people who work in the service. Those who may be a bit cautious and those who are not. And if I then integrate them at some point, it is as if I would be making a daily visit. And if there’s then no feedback, well, then all will be well.”* (GP: A/41).

Other GPs reported to find it difficult to hand over tasks for which they had to assume responsibility because they have neither professional nor disciplinary authority to give directives to nurses. In addition, some GPs have doubts about the competence of the nurse performing the task. On the other hand, nurses sometimes perceive having to consult the GP when the treatment must be changed as problematic and as complicating the care process.

GPs reported that they rarely use the option to supervise therapists for the handover of tasks:

*“[…], it happened only once so far that I didn’t really schedule an appointment with the doctor for her to watch me and then decide whether I was allowed to set the drain tube or not [.].”* (therapist: D/68).

#### Means of communication

The category comprises information about the means used by the participants to coordinate their work and to exchange information: by fax, phone, messenger services, e-mail, and by paper-based written documentation.

### Fax

Information mostly relates to non-urgent organisation, and health topics are primarily exchanged via fax between nurses, GPs, and therapists. Faxes are considered to mostly be effective, and nurses and therapists usually find the responses by the GPs appropriate:

*“This […] this is what we use most. The nursing service sends a fax that they need a new medication or that they need bandages, that they need this and that. And we send a fax back.”* (GP: A/37).

However, nurses mostly criticised that faxes are sometimes used in cases of sudden changes in health and for urgent concerns due to a lack of alternatives. Moreover, some nurses expressed frustration about not knowing whether the information they send reaches the GP, and they experience delays or get no response at all. To ensure proper care, they send reminders that, in the end, increase their workload:

*“Or they don’t even react to a fax […] that the entry points in which the PEG was inserted are inflamed, that this needs to be treated. That the GP has to come for a house call […] and it pisses me off pretty much when I have to attend to something five times before it gets done. That is very stressful, too.”* (nurse: A/17).

To simplify the process and to save time, some nurses and therapists reported that they send prepared fax templates.

### Phone

The phone is commonly used to exchange highly relevant or urgent information. Nurses and therapists reported many changes in the condition of PRHC, such as symptom deterioration via phone, to the GP practice. This was also noted by relatives. Communication via phone is usually perceived as unsatisfying, time-consuming, and as potentially slowing the care process down when the recipient (GP or nurse) is not available:

*“Making a phone call to the nursing services is frequently a problem because […] you typically don’t speak to the person who is familiar with the patient. And that is frequently unsatisfactory.”* (GP: A/37).

*“I make a call to the GP practice, the colleague is busy with another patient, I leave a message for him to call me back. He calls me back. I’m busy with another patient. I call him back. This can go on for the whole day. And that is an incredibly frustrating procedure, which makes you say in the end (with a sigh), ‘I just give up’.”* (therapist: A/108).

Some GPs clarified that they share their mobile number with professionals or sometimes the PRHC for a quick oral contact or an exchange via messenger service, particularly during out-of-practice hours, or when the health of the PRHC is expected to deteriorate:

*“[…], so that I offered my private telephone number for those I trust, where they can just send a text […], this is quite helpful on occasion.”* (GP: D/37).

The relatives stated that they also sometimes communicate via messenger app with the GP. A relative added that he/she also seek the guidance of nurses in advance to ensure that they use the correct wording.

### E-mail

E-mails play only a minor role in communication and are used for special contents such as photos or blood glucose levels. Furthermore, participants stated that e-mails are often considered to be troublesome and a waste of time by GPs for themselves or the staff in their practice:

*“And what is increasingly taking hold with us now is actually E-mail. Just now, I received the current blood sugar values from a patient who is suddenly dropping a bit: ‘I have taken a photo for you, I am sending it to you now, please tell me how I should proceed with this.”* (GP: C/61).

### Written documentation

Participants explained that a shared written documentation for professionals does not formally exist in the home care setting. This often results in parallel documentation systems. A nursing documentation folder contains written information for intraprofessional exchange. While some GPs criticised the inconsistent form and the rudimentary documentation in these folders, a few GPs or therapists reported that they also use it to obtain information or to document something, even though no space is provided for this. The PRHC said that they are sometimes aware of this interprofessional exchange.

Therapists pointed out that they very rarely have to write therapy reports upon request by GPs. Some therapists indicated that they proactively send reports to GPs to provide information. In rare cases, the GPs respond, but the therapists are frequently uncertain whether their communication has even reached the GP:

*“Well, there’s no feedback from any party, and I’m not quite sure where this all ends, in Neverland.”* (therapist: CB/79).

Conversely, some GPs complained about insufficient content of therapists` reports. This causes them to just quickly skim these reports or to not read them at all. The PRHC reported that they are generally aware of the existence of therapy reports, but not aware about their specific content.

In addition, persons of all groups described their developed informal documentation alternatives to coordinate or exchange information. Therapists write notes *“[…] we leave a note on a scrap of paper or something […].“* (therapist: D/50), or they create a “*handover book*” that is not used in the end. Nurses supplement their folder with a self-designed “*therapy sheet*” (nurse: A/188) to give therapists the opportunity for documentation.

Even relatives reported that they react to difficulties in coordinating individual solutions:

*“[…], so, they typically have a weekly calendar or a board at my place. It works quite well in this way. Then you know when ergo, physio, and logo [therapy] are due, whoever comes next.”* (therapist: A/32).

### Barriers and facilitators of interprofessional collaboration

Data revealed that person-related and structural factors influence interprofessional collaboration. We further describe the category “barriers and facilitators of interprofessional collaboration” within the following subcategories.

### Person-related factors

#### Being known

Participants of all groups perceived that knowing each other improves healthcare at home. Personal exchange, best in person, that is, in random encounters during visits at the PRHC`s place, lead to an intensive exchange, and this is viewed as enriching:

*“Well, when our nursing staff is there at the same time the physiotherapists are […], then of course they’ll discuss this and talk things over, about what’s going to be best for the patient […].”* (nurse: B/62).

The professionals stated that these encounters often serve as cornerstones for trust in each other’s expertise, resulting in closer working relationships and in optimising the care for PRHC. According to a therapist, even a single face-to-face encounter leads to more trust and open conversations:

*“If you happened to meet in person, nurse and physiotherapist, that is, you’d even agree on the way forward by discussing this among yourselves from that point on.”* (therapist: CB/94).

Many nurses and therapists expressed that contacting is easier when they have met the GP before. Moreover, the GP staff deal more quickly with concerns or put callers directly through to the GP when they are familiar with the nurses. In contrast, not “being known” by the practice staff might result in an unsatisfactory working relationship:

*“It is really quite different from when you don’t know them. You can get brushed off quite brusquely at times.”* (nurse: D/55).

“Being known” means that some GPs will share their private phone number to facilitate a closer exchange, which is favoured especially by small GP practices and home service providers, as they reported. Furthermore, some GPs stated that familiarity and trust is promoted by quarterly joint home visits with nurses. Although these visits do not cover costs and are deemed to be less effective, they are perceived as satisfying by nurses and GPs. Moreover, GPs emphasised that a strong mutual acquaintance with therapists has advantages:

*“[…] and at those medical practices where the contact is good, the therapy reports are good as well.”* (GP: D/57).

In consequence, some GPs stated that they recommend certain therapy practices. One nurse reported that she even introduces her home care service to surrounding GPs in person to initiate “being known”, and also to establish an expansion of her service.

Professionals reported that having contact persons in the GP practices and in the home care service who remain the same over time intensifies “being known”, resulting in increased trust in the other’s competences. One nurse complained that this trust is sometimes exploited by GPs to downplay the intensity of PRHC care. In consequence, nurses end the working relationship with these GPs because they feel that they are not being correctly informed or even misused. Many nurses reported to know in advance whether the collaboration would work depending on which GP was involved.

Some GPs admitted that not “being known” led to a lack of trust and resentment:

*“It always makes me really angry. ‘cause I think, what’s this again now? Don’t know a single name […]. I can’t really assess this*.” (GP: A/71).

#### Recognition of expertise

The mutual recognition of expertise is considered to be essential for a well-functioning collaboration. Many GPs told us that they appreciate nurses´ daily visit and their valuable feedback. Some GPs pointed out that they rate the quality of care as nurse-dependent. For nurses as one nurse reported, a professional presentation is essential for gaining respect and recognition of their expertise:

*“[…], respect is always gained through professionality. When I talk with a doctor, they need to realise that I know what I speak of. Then only will I be respected in turn.”* (nurse: A/25).

However, some nurses and therapists stated that GPs lack awareness of the expertise the nurse has. On the other hand, therapists reported an interest by GPs as they respond to therapy reports, and a closer relationship develops following that report. Furthermore, the expertise of GPs was criticised by nurses, who complained that the knowledge of the GP in prescribing options is inadequate. In addition, many nurses and therapists complained about a lack of recognition of their expertise by the respective others. Therapists observed that nurses sometimes feel downgraded when instructed in treatment options (e.g. in positioning techniques). Nurses sometimes considered the assistance as an additional workload rather than a facilitation, and are often not interested.

### Structural conditions

The subcategory comprises several further categories: availability, financial and time constrains, and fragmentation of care.

#### Availability

The GPs and therapists reported that they sometimes face challenges in reaching the responsible nurse by phone, which leads to repeated and exhausting attempts. This often results in constant new attempts at contacting the other professionals to establish collaborative working relationships. Large home service providers are better available and facilitate information transfer. Moreover, nurses complained that they have problems to inform GPs because they are not directly available or because there is no dedicated contact person in the GP’s practice. An increased workload for contact attempts arises, and safe care is more difficult to deliver. However, nurses stated that they often experience good availability of GPs in an urgent situation, which finally ensures high-quality care. All professionals considered the availability of therapists to be a challenge because of the different working-time models and divergent practice organisations.

#### Financial and time constraints

Participants of all groups noted that financial and time constraints influence a collaborative practice. Generally, interprofessional collaboration needs time, which is not left on the professional side and is also not remunerated.

#### Fragmentation of care

Too many professionals, too much communication, and too many care processes might implicate limitations in the quality of care. Nurses describe their worries about the lack of a dedicated contact person making safe care more difficult, especially in practices with several GPs. GPs complained that the many therapists’ offices and home care services in their vicinity prevent them from effectively interacting with them. Additionally, too many or only temporarily assigned nurses result in repeated new onboarding by the GP:

*“[…] It’s rather annoying about bandages at times […], when the nursing service sends a different person every day, meaning that this person needs to familiarise themselves a new.”* (GP: D/33).

Moreover, therapists stated that changes of nurses, care routes, or shifts cause agreements to lapse. Many GPs reported also that they fear the default of agreements by nurses because of the immense lack of staff and high fluctuation rates:

*“[…], or you agree on something […] that is not met the next day because that person needs to help out somewhere else.”* (therapist: A/76).

At least participants spoke about good experiences with interprofessional exchange when home service providers or therapists are responsible for all PRHC of one GP. Finally, some nurses and GPs stated that they have difficulties finding therapists providing therapy at home at all.

## Discussion

### Principal findings

The present study explored the perception of interprofessional collaboration in the home care setting among PRHC, relatives, nurses of home care services, GPs, and members of therapy professions. Three main categories evolved: “perception of interprofessional collaboration”, “means of communication”, and “barriers and facilitators”. Different aspects of collaboration were uncovered, such as organisational coordination, information exchange, and complementary collaboration. The intensity of the collaboration seems to differ in all categories according to the medical, nursing, therapeutic, and psychosocial complexity of the healthcare situation. The most frequent means of communication are fax and phone, which are both sometimes considered as unsatisfying and time-consuming. As little formal written or electronic documentation systems exist, some participants create their own systems. Person-related factors such as knowing each other and mutual recognition of expertise were considered crucial for an effective collaboration. However, reliable structures that would ensure the availability of other professionals were difficult to establish. Moreover, structural factors (financial and time constraints) affect the collaboration. The fragmentation of care contributes to concerns about the quality and continuity of care.

### Findings compared to other studies and literature

This study reveals that interprofessional collaboration is rare. In less complex care situation, the participants often do not perceive this as relevant or necessary. Our findings are in line with those in previous international studies in the homecare setting, which stated that professionals mostly work independently of each other and often do not perceive the need to change the current care structures or recognize the potential benefits of collaboration [6, 7, 10]. Specifically, the expertise of occupational therapy is unknown to nurses, GPs or even physio therapists [6, 7, 10]. PRHC and relatives do not perceive a need for change either, are still mostly satisfied with their healthcare provision, and may not even be aware of interprofessional collaboration at all [16].

Our findings indicate that organisational coordination is often the only contact between professionals and is perceived as challenging. Overlapping appointments, loss of therapy time, and cancellations are negative consequences of coordination failures. This has also been revealed by the following studies: Sakai et al. suggested in their findings that well-coordinated teams are more effective at meeting client needs than poorly coordinated teams [24]. Notably, the lack of passing on information about new prescriptions or changed orders is potentially a high risk in the care process [25]. It can be concluded that connections between professionals allow a reliable exchange of information and a fast feedback that promotes organisational coordination and therapy adjustments in home care.

Our results show that information exchange is perceived as essential for collaboration, especially in complex care situation, but it is not formally settled. This correlates with other international studies that reported that collaboration works sufficiently in some parts, but is formally unresolved [10, 11, 25–28]. The lack of communication rules for contact initiative, timing, content, and standardised means is seen as critical in the current care structures [25]. Inadequate information sharing with the PRHC or relatives, who often serve as mediators, poses significant risks for adverse events and errors in care [5, 9, 10, 16, 25, 27, 29]. However, according to our data, some PRHC and relatives expressed an explicit desire to be integrated in and keep control of the care situation. Nurses often take over as mediators from PRHC or relatives and hold significant responsibility for the PRHC. Several studies [7, 16, 30, 31] also stated that nurses shoulder the main collaboration effort. This might imply that in less complex care situations in which the various health carers are not connected in advance, a sudden onset of a health crisis or a failure of one professional or the relatives to contact the other professionals and/or relatives can lead to a vulnerable situation [16].

As indicated by our data, professionals are more often in contact and support and meet each other to a greater extent when an individual care situation becomes more complex or when the health status of a patient appears to be about to deteriorate. Similar findings have been reported by other authors [3, 7, 10, 16, 27, 31–33]. Moreover, according to the framework of Careau et al., collaborative practices, such as the intention to build a partnership with clients or their families, interaction between practitioners, and the combination of disciplinary knowledge, should intensify according to the increasing complexity of the biological, psychological, and social needs of the client [14]. Here, interprofessional connectivity could alleviate workloads and provide more flexible care by combining medical and nursing tasks for continuous adjustments in the case of growing care needs [3, 26, 34].

Another key topic is the difficulty of choosing the most expedient means of communication. This leads to inconsistencies and frustration among healthcare professionals. The literature agrees with this and states that the lack of shared formal documentation systems and of reliable means of communication might result in double documentation, incomplete information, poor mutual availability, and insufficient communication [3, 8, 10, 31, 34]. According to our data, direct contact between professionals is rare in general. On the one hand, this might be partially explained by missing formal communications structures, poor availability of the other professionals, financial and time constraints, and by fragmentation of care. On the other hand, there might be no perceived need for it, especially in less complex healthcare situations.

Our data also support the idea that interprofessional collaboration is influenced by person-related factors, trust, and by a mutual appreciation of the expertise of the various participants in the care. This is consistent with several studies in which the participants emphasised that it was important to be known and trusted to foster effective communication and collaboration in interprofessional healthcare settings [7, 10, 25–27, 29, 31, 35]. Interprofessional training can offer professionals the opportunity to acquire competencies such as knowledge, skills, attitudes, and behaviours that empower them to engage in collaborative work and in a shared vision of a more patient-centred responsive care [35, 36].

Additionally, our results with regard to barriers for interprofessional collaboration are supported by several studies indicating a lack of time and inadequate reimbursement [3, 26, 31]. Our data also reaffirm the challenges professionals face in the form of differing work schedules and processes, geographic boundaries, and staff fluctuation [5, 8, 29, 31, 33, 37]. The attempts of GPs to minimise communication with home care services by encouraging the PRHC or relatives to select their (the GP’s) preferred service was reported also by Nieuwbower et al. [31]. Secure audio-conferences as a way to increase care planning for shared patients were held in the study of Berg et al., who reported mixed experiences such as miscommunication and late participants, but also efficient communication and a more comprehensive picture [8].

### Strengths and limitations

The greatest strengths of our study are the different person groups that are covered by our sample, and therefore the heterogeneity of the sample with regard to the variables such as geographical localisation, care needs, and the role of the relatives involved. To the best of our knowledge, no study of the perceptions of interprofessional collaboration that included PRHC, relatives, nurses, GPs, and therapists has been published so far. Thus, our study provides a comprehensive view of healthcare at home in Germany. Another strength is the diverse professional backgrounds of the study team. The wide range of perspectives we brought to the study helped us to evaluate and discuss the data, and we were able to generate detailed and complex findings. All researchers were trained with regard to the procedures of recruitment, informed consent, data collection, qualitative data analyses, and data security and ethics by AM, BT, and CM.

Our study contains a few limitations: a selection bias can be assumed because PRHC were interviewed by telephone, which might implicate the physical and mental ability to handle challenging technical conditions and the duration of the interviews. By interviewing relatives, we obtained insights into complex care situations and bridged the gap of this possible limitation. While recruiting, we did not differentiate between relatives that lived at a distance and those that lived close by. This might have shown finer gradations in the extent of the perceived collaboration. Another limitation arose because data were collected during the Covid-19 pandemic via phone (interviews) and video conference (focus groups) because of distance constraints. In consequence, the human conjunction of qualitative research may be missing. However, Farooq and de Viliers identified criteria for phone interviews that matched our experience: interviewees and interviewers had no difficulties to conduct phone interviews, and the context of the home environment was not relevant for our questions [38]. Yom et al. showed that the statements in video-based and in-person focus groups overlap in content [39]. The final possible limitation is related to the sample of professional participants. Our study was conducted in the German home care setting, where the GP practices we recruited as well as the home care service providers and therapist practices work mostly independently of each other in different locations. This might limit the transferability of the results to other settings for example nursing homes as well as to other countries.

## Conclusion

Our study is innovative as it analysed the perceptions of interprofessional collaboration of different groups of professionals, PRHC and relatives to receive a comprehensive view, and broad, differentiated understanding of the home care setting in Germany. It contributes to the expanding literature on interprofessional collaboration in home healthcare. Currently, interprofessional care rarely takes place in the healthcare situations of PRHC. Organisational coordination and information exchange often lie in the hands of the PRHC or relatives, and it does not always work sufficiently. The home care situation might become fragile because collaboration processes between professionals are not predefined for a worsening in the health of the PRHC.

In more complex situations, professionals work more closely together. Joint meetings, potentially as home visits, and a designated coordinating person within the care team could help to establish clear structures and a “being known” right from the start of the care situation. The integration of a common digital tool encompassing documentation and communication could enhance care continuity and timely response for further action. With the realisation that the perception differs between the involved persons groups, tailored interventions might better meet the needs.

A better interprofessional collaboration might enable the anticipation of adverse events and bridge gaps in the home health care situation. According to van den Bussche et al. the avoidance of hospital admissions is estimated more optimistically when the collaboration is good [10]. Interprofessional collaboration is also a sensitive topic in other countries regarding the impact of good health care at home [5–8, 16, 29–32, 34, 35, 40].

Our findings might serve as a base for future research focusing on the development of interprofessional person-centred interventions to optimise quality of care in the home care setting.

### Electronic supplementary material

Below is the link to the electronic supplementary material.

Supplementary Material 1: Guidelines for interviews with PRHC and relatives

Supplementary Material 2: Guidelines for monoprofessional focus groups with nurses, GPs and therapists

## Data Availability

Transcripts are held in secure, confidential, password-protected storage at the Department of General Practice at the University Medical Center Göttingen, the Department of General Practice and Primary Care, University Medical Center Hamburg-Eppendorf, the Nursing Research Unit of the University of Lübeck, and the Institute of Nursing Science of theUniversity of Cologne. Access to data cannot be provided as the consent forms state exclusive use by the study team for study purposes. Original data therefore cannot be shared.

## Footnotes

1 Identifiers: The first term denotes the person group, the capital letters stand for the research centres (A, B, C, D). In case of interviews, the following number indicates the number of the interviewee, and the numbers behind the slash refer to the line in the transcript.

## Abbreviations

GP: General practitioners
PRHC: People receiving home care
WHO: World Health Organization
