# Supplementary material for: Interprofessional collaboration in the home care setting: perspectives of people receiving home care, relatives, nurses, general practitioners, and therapists—results of a qualitative analysis
Source: BMC Prim Care. 2024 Mar 4;25:79. doi: 10.1186/s12875-024-02313-8 (PMC10910757; doi:10.1186/s12875-024-02313-8)
Supplement: Supplementary file 1 — Supplementary Material 1: Guidelines for interviews with PRHC and relatives [file 12875_2024_2313_MOESM1_ESM.docx]

**Additional Material-Additional file 1-Guidelines to open interviews**

Guidelines to open interviews with persons receiving home care

| Report the current care situation | What does a typical day look like for you when you think of your healthcare situation? Please tell us about it. |
| --- | --- |
| Experience with collaboration of the different professionals | You are being cared for by many different persons of various professions {list the persons according to the professions}. Would you tell us how they collaborate, in your view?  If you had to be hospitalised recently, can you tell us who was involved in that? Who decided you needed to be hospitalised, and how was it all managed?  What was the situation like when you were released to go home again? Would you please tell us how that was managed?  The Covid-19 pandemic and the measures that had to be taken to counter it probably changed the way you received your care compared with before the pandemic, for instance in the collaboration |
| person centred/inclusion of relatives and trusted friends | In which way are you involved in your own care (any exchange of information)?  In which way are your relatives or trusted friends involved in your own care (any exchange of information)? |
| Ideas and golden dreams about what an ideal home healthcare would look like | If you were to develop a golden dream about what the ideal home healthcare would look like, what would be on your mind? What would be ideal for your situation at home? |

Guidelines to open interviews with relatives

| Report the current care situation | What does a typical day look like for you when you think of the healthcare situation of your … (father, mother, aunt etc.)? Please tell us about it. |
| --- | --- |
| Experience with collaboration of the different professionals | Your …is being cared for by many different persons of various professions {list the persons according to the professions}. Would you tell us how they collaborate, in your view?  If your …had to be hospitalised recently, can you tell us who was involved in that? Who decided that your …needed to be hospitalised, and how was it all managed?  What was the situation like when your …was released to go home again? Would you please tell us how that was managed?  The Covid-19 pandemic and the measures that had to be taken to counter it probably changed the way your …received care compared with before the pandemic, for instance in the collaboration |
| person-centred/inclusion of relatives and trusted friends | In which way are you or your …involved in the care of your … (any exchange of information)? |
| Ideas and golden dreams about what an ideal home healthcare would look like | If you were to develop a golden dream about what the ideal home healthcare would look like, what would be on your mind? What would be ideal for your situation at home? |
