# Supplementary material for: Interprofessional collaboration in the home care setting: perspectives of people receiving home care, relatives, nurses, general practitioners, and therapists—results of a qualitative analysis
Source: BMC Prim Care. 2024 Mar 4;25:79. doi: 10.1186/s12875-024-02313-8 (PMC10910757; doi:10.1186/s12875-024-02313-8)
Supplement: Supplementary file 2 — Supplementary Material 2: Guidelines for monoprofessional focus groups with nurses, GPs and therapists [file 12875_2024_2313_MOESM2_ESM.docx]

| Report your own experience with interprofessional collaboration in the context of people receiving home care | We now turn to your own experience with interprofessional care of people receiving home care.  We would like you first to report your own interaction or direct contact with the relatives of people receiving home care or with trusted friends of these persons.  Have you had personal experience so far in collaborations with general practitioners in providing care to people receiving home care?  Please also relate your personal experience of working with occupational therapists, physiotherapists, and/or speech therapists in providing care to people receiving home care.  Some of you appear so far not to have had personal experience of a collaboration with other professionals in home care. What might be the reasons for this?  In which way do you involve people receiving home care and their relatives or trusted friends when you communicate with professionals of other special fields? Do you involve them in the decision-making process, and if so, how do you involve them?  Did Covid-19 change the existing collaboration in any way? |
| --- | --- |
| Formulating ideas to optimise collaboration with other professionals who are part of the care team | We would like to take half an hour now to formulate ideas together how a future person-centred interprofessional collaboration could look like when the other professionals who are part of the care team are involved and also the people receiving home care and their relatives or trusted friends.  Please let us know how you envisage a collaboration with other professionals of the care team.  So far, we’ve mostly talked about a collaboration with general practitioners/therapists. How could people receiving home care and their relatives or trusted friends be included in this?  Finally, we would like to take another general pass: Is there anything that we’ve not touched upon so far, but that you consider particularly important? |

**Additional Material – Additional file 2-Guidelines for the monoprofessional focus groups**

Guidelines for the monoprofessional focus groups with nurses

| Report your own experience with interprofessional collaboration in the context of people receiving home care | We now turn to your own experience with interprofessional care of people receiving home care.  We would like you first to report your own interaction or direct contact with the relatives of persons receiving home care or with trusted friends of these persons.  Have you had personal experience so far in collaborations with nurses from home care services in providing care to persons receiving home care?  Please also relate your personal experience of working with occupational therapists, physiotherapists, and/or speech therapists in providing care to persons receiving home care.  Some of you appear so far not to have had personal experience of a collaboration with other professionals in home care. What might be the reasons for this?  In which way do you involve persons receiving home care and their relatives or trusted friends when you communicate with professionals of other special fields? Do you involve them in the decision-making process, and if so, how do you involve them?  Did Covid-19 change the existing collaboration in any way? |
| --- | --- |
| Formulating ideas to optimise collaboration with other professionals who are part of the care team | We would like to take half an hour now to formulate ideas together how a future person-centred interprofessional collaboration could look like when the other professionals who are part of the care team are involved and also the persons receiving home care and their relatives or trusted friends.  Please let us know how you envisage a collaboration with other professionals of the care team.  So far, we’ve mostly talked about a collaboration with home care services, occupational therapists, physiotherapists, and/or speech therapists. How could persons receiving home care and their relatives or trusted friends be included in this?  Finally, we would like to take another general pass: Is there anything that we’ve not touched upon so far, but that you consider particularly important? |

Guidelines for the monoprofessional focus groups with general practitioners

Guidelines for the monoprofessional focus groups with therapists

| Report your own experience with interprofessional collaboration in the context of people receiving home care | We now turn to your own experience with interprofessional care of people receiving home care.  We would like you first to report your own interaction or direct contact with the relatives of people receiving home care or with trusted friends of these persons.  Have you had personal experience so far in collaborations with general practitioners in providing care to people receiving home care?  Please also relate your personal experience of working with nurses from home care services in providing care to people receiving home care.  Some of you appear so far not to have had personal experience of a collaboration with other professionals in home care. What might be the reasons for this?  In which way do you involve people receiving home care and their relatives or trusted friends when you communicate with professionals of other special fields? Do you involve them in the decision-making process, and if so, how do you involve them?  Did Covid-19 change the existing collaboration in any way? |
| --- | --- |
| Formulating ideas to optimise collaboration with other professionals who are part of the care team | We would like to take half an hour now to formulate ideas together how a future person-centred interprofessional collaboration could look like when the other professionals who are part of the care team are involved and also the people receiving home care and their relatives or trusted friends.  Please let us know how you envisage a collaboration with other professionals of the care team.  So far, we’ve mostly talked about a collaboration with general practitioners and nurses. How could people receiving home care and their relatives or trusted friends be included in this?  Finally, we would like to take another general pass: Is there anything that we’ve not touched upon so far, but that you consider particularly important? |
